# ContransGAN: Convolutional Neural Network Coupling Global Swin-Transformer Network for High-Resolution Quantitative Phase Imaging with Unpaired Data

**DOI:** 10.3390/cells11152394

**Published:** 2022-08-03

**Authors:** Hao Ding, Fajing Li, Xiang Chen, Jun Ma, Shouping Nie, Ran Ye, Caojin Yuan

**Affiliations:** 1 Key Laboratory for Opto-Electronic Technology of Jiangsu Province, Nanjing Normal University, Nanjing 210023, China; 191035004@njnu.edu.cn (H.D.); 191002029@njnu.edu.cn (F.L.); 211002001@njnu.edu.cn (X.C.); nieshouping@njnu.edu.cn (S.N.); ran.ye@njnu.edu.cn (R.Y.); 2 School of Electronic Engineering and Optoelectronic Techniques, Nanjing University of Science and Technology, Nanjing 210094, China; majun@njust.edu.cn

**Keywords:** super-resolution, transformer, CNN, quantitative phase imaging, transport of intensity equation

## Abstract

Optical quantitative phase imaging (QPI) is a frequently used technique to recover biological cells with high contrast in biology and life science for cell detection and analysis. However, the quantitative phase information is difficult to directly obtain with traditional optical microscopy. In addition, there are trade-offs between the parameters of traditional optical microscopes. Generally, a higher resolution results in a smaller field of view (FOV) and narrower depth of field (DOF). To overcome these drawbacks, we report a novel semi-supervised deep learning-based hybrid network framework, termed ContransGAN, which can be used in traditional optical microscopes with different magnifications to obtain high-quality quantitative phase images. This network framework uses a combination of convolutional operation and multiheaded self-attention mechanism to improve feature extraction, and only needs a few unpaired microscopic images to train. The ContransGAN retains the ability of the convolutional neural network (CNN) to extract local features and borrows the ability of the Swin-Transformer network to extract global features. The trained network can output the quantitative phase images, which are similar to those restored by the transport of intensity equation (TIE) under high-power microscopes, according to the amplitude images obtained by low-power microscopes. Biological and abiotic specimens were tested. The experiments show that the proposed deep learning algorithm is suitable for microscopic images with different resolutions and FOVs. Accurate and quick reconstruction of the corresponding high-resolution (HR) phase images from low-resolution (LR) bright-field microscopic intensity images was realized, which were obtained under traditional optical microscopes with different magnifications.

## 1. Introduction

Recent advances in microscopy have allowed imaging of biological processes with a higher level of quality [1,2,3,4]. However, these microscopy techniques are usually limited by sophisticated setups and special experimental conditions. In addition, the resolution of optical microscopic imaging is limited by the numerical aperture (NA) of the microscope, the wavelength of illuminating light, and the pixel spacing of the imaging recording device. As a result, researchers in related fields are usually committed to dealing with the above trade-off and improving the imaging efficiency [5].

Since the huge volume of data and the continuous improvement of the computing power, artificial intelligence (AI) has developed rapidly. Over the past few years, deep learning technology has been leading the development of AI and is widely used in computer vision [6], natural language processing [7], speech recognition [8], and other fields. Deep learning uses multiple-layer neural networks to automatically analyze signals or data, which has unique advantages in solving “inverse problems” and nonlinear problems. The unique advantages of deep learning technology have also aroused the interest of optical imaging scholars. Deep learning technology has been used to solve problems that lack effective solutions in optical computational imaging, such as optical tomography [9,10], optical fiber imaging [11], ghost imaging [12], scattering imaging [13], and low-light environment imaging [14]. At the same time, deep learning technology has also been widely used in various important directions of QPI, such as phase retrieval [14,15,16,17,18], super-resolution [19,20,21,22,23], phase unwrapping [24,25,26,27], label-free detection [28], and various other image enhancement techniques [29,30,31,32]. There is no exception for the use of deep learning in breaking through the trade-off between the resolution and the field of view in microscopy [33,34]. The general idea is to use a network to learn the relationship between LR and HR image pairs. The input LR image will be converted into an HR image but the same field of view as an LR image is maintained using the built network model. However, the required pairs of LR-HR data are obtained by mechanically switching between the low NA and high-power objectives in the experiment and numerical registration in pretreatment, which is a very tedious process. In order to replace the time-consuming image registration processes, Zhang [35] et al. degraded the captured HR microscopic images to simulate the LR microscopic image, However, it is necessary to adjust the parameters repeatedly to ensure that it is similar to the LR images, which are obtained by the experiment. In addition, the simulated LR microscopic images are not consistent with the real image degeneration process, which usually includes blurring, noise, and other defects. Further, some works [36,37,38] proposed the use of unsupervised methods for super-resolution imaging, in order to reduce the amount of training data. The feature extraction results of unsupervised methods are random to some extent, so noise or blurring is easily amplified. Meanwhile, there are some works that train the network using unpaired datasets [39,40]. This kind of semi-supervised deep learning network framework greatly reduces the difficulty of datasets acquisition. However, these works only involve conversions between image styles (such as intensity images are converted to phase images) and do not simultaneously achieve image resolution enhancement due to the traditional CNN network showing a poor performance in multiple tasks. For example, it is difficult to achieve the expected imaging effect when performing super-resolution and quantitative phase imaging at the same time [41].

Researchers have attempted to improve the network performance by adding the residual module, feedback mechanism, or attention mechanism [42,43,44] to CNN. However, these frameworks still have some fundamental limitations. On the one hand, the convolution operation of CNN is good at extracting local features but poot at extracting global features. The lack of a global understanding ability results in a loss of rich information in LR microscopic. On the other hand, the weights of the convolution network are fixed and it cannot adapt to the change in the input dynamically.

In this paper, we propose an end-to-end deep learning framework, which is termed ContransGAN, coupling CNN with Vision Transformer (ViT) [45] to obtain HR phase information from LR intensity information. This framework retains the advantage of CNN in extracting the details of local features, and enhances its ability to capture the global feature. Furthermore, the network framework can be trained with unpaired images, which ease the experiment and data preparation. The large FOVs HR quantitative phase images can be reconstructed from the LR intensity image using the trained ContransGAN. We verified the effectiveness of the ContransGAN algorithm and its generalization performance by acquiring LR microscopic images of different samples under an inverted microscope.

## 2. Methods

### 2.1. Imaging Hardware and TIE Phase Extraction

TIE is an ideal candidate for phase imaging with partially coherent illuminations [46]. In relation to its closed-form solution, TIE offers a cost-efficient way of measuring the phase by a single step. The phase recovery by TIE is standard and is briefly explained for the completeness of this paper. In the paraxial approximation, TIE can be expressed as the following format

(1) $$-k\frac{\partial I\left(r\right)}{\partial z}=\nabla \cdot \left[I\left(r\right)\nabla \varphi \left(r\right)\right]$$

where k=2π/λ is the wave number, r represents the transverse spatial coordinates perpendicular to the optical axis, φ is the phase distribution of the sample, ∇ is the two-dimensional gradient with respect to r, and $I\left(r\right)$ represents the actual intensity distribution along the optical axis in the plane of z=0. The left-hand side of the Equation (1) represents the axial differentiation of the intensity distribution. Solving the TIE requires the axial differentiation to be obtained in advance. Specifically, the axial differentiation of the intensity distribution is obtained by acquiring two slightly out-of-focus images with an equal off-focus distance and opposite direction with respect to the center-focused image, using the central finite difference method estimation [47]. As expressed in Equation (2), the derivative of the intensity distribution is approximated by the finite difference as

(2) $$\frac{\partial I\left(r\right)}{\partial z}\approx \frac{I\left(r,∆z\right)-I\left(r,-∆z\right)}{2∆z}$$

and the extracted phase can be expressed as the Equation (3)

(3) $$\varphi \left(r\right)\approx -k\nabla^{-2}\nabla \cdot [I^{-1}\left(r\right)\nabla \nabla^{-2}\frac{I\left(r,∆z\right)-I\left(r,-∆z\right)}{2∆z}$$

where $\nabla^{-2}$ is the inverse Laplacian operator. In our work, we solved the TIE using the fast Fourier transform (FFT) algorithm [48] under the homogeneous Neumann boundary condition [49].

**Figure 1:**
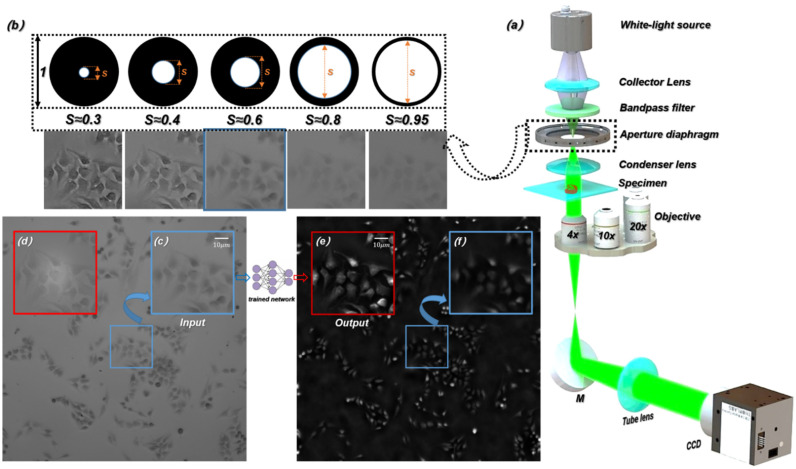
Experimental setup and acquisition process of the original images. (**a**) Imaging hardware setup. (**b**) Microscopic images obtained under a different aperture diaphragm. (**c**) The LR microscopic image captured directly in the experiment. (**d**) The HR microscopic image captured directly in the experiment. (**e**) The reconstructed HR quantitative phase image by TIE. (**f**) The reconstructed LR quantitative phase image by TIE.

As shown in Figure 1a, an inverted microscope (Nikon ECLIPSE Ti-S) was used for the experimental setup. The illumination light source was a halogen lamp, and the central wavelength of the filtered illumination light source was 550 nm. The beam passed through the specimen and carried the specimen’s information, and then is focused by an objective. The microscopic images were captured by a CCD camera through a tube lens. Under the high-power microscope objective (40×/0.65 NA), we captured the microscopic images with the same out-of-focus depths (∆z=3 μm) on both sides of the focal plane of the specimen and the corresponding in-focus microscopic images, and then extracted the corresponding HR phase information with the TIE algorithm [50,51,52,53]. Under the low-power microscope objective (4×/0.1 NA, 10×/0.25 NA, and 20×/0.4 NA), we captured LR microscopic images with different resolutions as the input of the ContransGAN.

In our experiment, the spatial coherence of the illumination light source is determined by the size of the aperture diaphragm [54]. The spatial coherence is represented by the coherence parameter S, which is the ratio of the condenser aperture to the objective NA. As shown in Figure 1b, the contrast and resolution of the images that are recorded under the corresponding aperture diaphragm are different. Adjusting the aperture of the diaphragm to S≈0.3 ensures that the captured microscopic images have fine contrast, so that quantitative phase information can be calculated by TIE [46,55]. Meanwhile, adjusting the aperture of the diaphragm to S≈0.6 captures the LR microscopic images. Figure 1c,f show the microscopic image obtained in the experiment and the corresponding LR phase image recovered by TIE respectively. Figure 1d,e show the HR intensity image and the HR quantitative phase image reconstructed by TIE respectively. The proposed deep learning framework finally generates the HR phase images consistent with the Figure 1f.

### 2.2. Creation of Datasets and Networks Training Details

**Figure 2:**
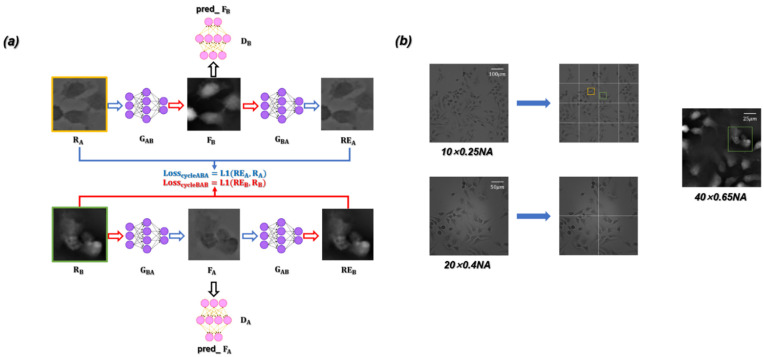
(**a**) Training flow diagram. (**b**) By segmenting the LR microscopic images with different resolutions, the sub-images with an FOV approximately equal to the HR quantitative phase images are obtained for training the ContransGAN. Orange and green rectangles are Region of Interest (ROI).

The ContransGAN proposed in our work was designed based on the CycleGAN architecture [56]. The network framework is essentially composed of two symmetrical generative adversarial networks (GAN). The flow chart of the entire training is shown in Figure 2a. The framework includes the generator $G_{\mathrm{AB}}$ and generator $G_{\mathrm{BA}}$ for performing conversion between images. Correspondingly, the discriminator $D_{A}$ and discriminator $D_{B}$ are responsible for judging whether the images generated by the generators are close to the reality. The training dataset consists of $R_{A}$ (input, LR microscopic images) and $R_{B}$ (ground truth, HR quantitative phase images) respectively. During the process of training, the LR microscopic image in $R_{A}$ is input into the generator $G_{\mathrm{AB}}$ to obtain $F_{B}$, and then $F_{B}$ is input into the discriminator $D_{B}$ to extract eigenvalues, which are used to calculate ${\mathrm{Loss}}_{\mathrm{DB}}$. At the same time, $F_{B}$ is also input into $G_{\mathrm{BA}}$ to generate ${\mathrm{RE}}_{A}$. The training process of $R_{B}$ is consistent with that of $R_{A}$. As expressed in Equation (4), the overall loss function can be written as

(4) $$\mathrm{Loss}=\left[{\mathrm{Loss}}_{\mathrm{GAN}}\right]+\lambda \left\{{\mathrm{Loss}}_{\mathrm{cycle}}\right\}\;=\left[{\mathrm{Loss}}_{\mathrm{DA}}+{\mathrm{Loss}}_{\mathrm{DB}}\right]+\lambda \left\{{\mathrm{Loss}}_{\mathrm{cycleABA}}+{\mathrm{Loss}}_{\mathrm{cycleBAB}}\right\}$$

where λ is used to adjust the proportion of ${\mathrm{Loss}}_{\mathrm{cycle}}$, and the value is set to 10. The main function of ${\mathrm{Loss}}_{\mathrm{GAN}}$ is to mutually promote the performance of the generators and discriminators. The overall loss function enables the generators to produce images approximating well to real ones; the main function of ${\mathrm{Loss}}_{\mathrm{cycle}}$ is to ensure that the output images of the generators are different from the input images in style but consistent in content. Specifically, as expressed in Equation (5), ${\mathrm{Loss}}_{\mathrm{GAN}}$ can be written as

(5) $$\begin{array}{c} {\mathrm{Loss}}_{\mathrm{GAN}}={\mathrm{Loss}}_{\mathrm{DA}}+{\mathrm{Loss}}_{\mathrm{DB}}\\ =E_{b}\left[{\left(D_{B}\left(b\right)-1\right)}^{2}\right]+E_{a}\left[(1-D_{B}{\left(G_{\mathrm{AB}}\left(a\right))\right)}^{2}\right]\;\\ +E_{b}\left[{\left(D_{A}\left(a\right)-1\right)}^{2}\right]+E_{b}\left[(1-D_{A}{\left(G_{\mathrm{BA}}\left(b\right))\right)}^{2}\right]\; \end{array}$$

where $E_{\left[\cdot \right]}$ represents the expected value of the random variable in square brackets; a and b represent the images in dataset $R_{A}$ versus $R_{B}$ respectively. ${\mathrm{Loss}}_{\mathrm{cycle}}$ is expressed in Equation (6), which used to further optimize the model. It can be written as

(6) $$\begin{array}{c} {\mathrm{Loss}}_{\mathrm{cycle}}={\mathrm{Loss}}_{\mathrm{cycleABA}}+{\mathrm{Loss}}_{\mathrm{cycleBAB}}\\ =E_{a}\left[∥G_{\mathrm{BA}}\left(G_{\mathrm{AB}}\left(a\right)-a\right){∥}_{1}\right]+E_{b}\left[∥G_{\mathrm{AB}}\left(G_{\mathrm{BA}}\left(b\right)-b\right){∥}_{1}\right]\; \end{array}$$

where $∥\cdot {∥}_{1}$ represents the norm L1.

In this paper, unstained Hela cells and polystyrene microspheres (PSMs) are used as the experimental specimens. $R_{A}$ and $R_{B}$ consist of 3500 unpaired LR microscopic images and 3500 HR quantitative phase images respectively. It is worth noting that by segmenting the original LR microscopic images, we obtain the LR microscopic images, which are approximately equal to the FOVs of the HR quantitative phase images. As shown in Figure 2b, the LR microscopic image that was captured by the 10×/0.25 NA objective is equally divided into 16 sub-images, so that the field-of-view range of each sub-image is approximately equal to that of the 40×/0.65 NA objective. The FOV of each sub-image is approximately equal to the HR quantitative phase image reconstructed by the corresponding microscopic images captured under the 40×/0.65 NA objective. In the process of model building, in order to enhance the network generalization ability and improve the training efficiency and precision, we cropped or scaled the input LR microscopic images by random image interpolation [57]. Among all the datasets, 85% are used for the training dataset and the remaining 15% for the testing dataset. The ContransGAN is implemented by python 3.6.8 based Pytorch 1.3.1 and the network training and testing on a PC with double Intel Aeon Gold 5117 CPU @ 2.00 GHz and 128 GB RAM, using NVIDIA Forced RTX 2080 Ti GPU. The training process takes ~50 h for 80 epochs (in a batch size of 2). Finally, the imaging speed of the trained ContransGAN for a phase image can reach ~0.06 s.

### 2.3. Vision Transformer and Self-Attention Mechanism

**Figure 3:**
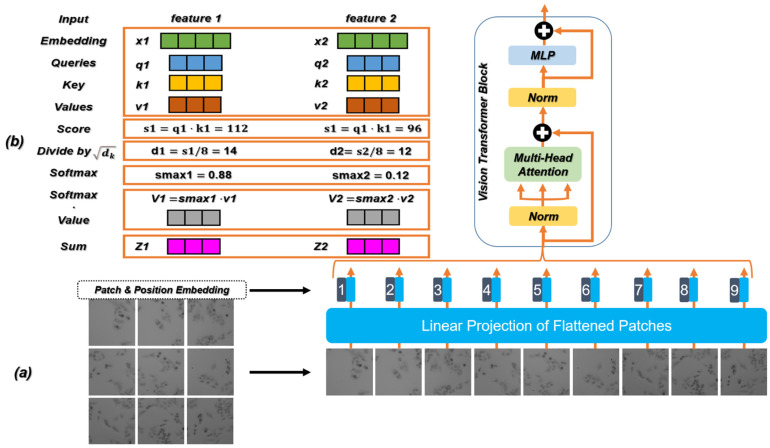
(**a**) The feature extraction process and the specific structure of ViT. (**b**) Calculation flow chart of the self-attention mechanism in ViT.

Before introducing the generator and the discriminator, it is necessary to introduce the relevant theories of the Transformer in detail. Transformer [58] is a classic model for natural language processing (NLP) proposed by Google in 2017. It uses the self-attention mechanism instead of the sequential structure of the recurrent neural network (RNN) [59] so that the model can be trained in parallel and has global information. Recently, the Transformer structure has been used in ViT. Figure 3a shows the part used for feature extraction in ViT, which constructs a series of marker sequences by dividing each image into Patch with position embedding, and then uses the Transformer module to extract parametric vectors as visual representations. Position embedding records the sequence correlation between sequence data. Compared with the characteristics of the RNN sequential input, the method based on Transformer can directly input data in parallel and store the position relationship between data, which greatly improves the computing speed and reduces the storage space. In addition, with the increase in the number of network layers, the distribution of the data will continue to change. In order to ensure the stability of the data feature distribution, a layer of regularization [60] is introduced to reduce information loss.

The attention mechanism imitates the internal process of biological observation behavior and enhances the fineness of observation in some areas. Since it can quickly extract the important features of sparse data, the attention mechanism is widely used in machine translation, speech recognition [61], image processing [62], and other fields. The attention mechanism has become an important concept in the field of neural networks. It is an advanced algorithm for multitasking, which is widely used to improve the interpretation of neural networks, and helps to overcome some challenges in RNN, such as performance degradation with the increase in the input length and computational inefficiency caused by an unreasonable input sequence. The self-attention mechanism is the improvement of the attention mechanism, which reduces the dependence of the network on external information and is better at capturing the internal relevance of data or features. Transformer introduces the self-attention mechanism to avoid the use of recursion in the neural network, and completely relies on the self-attention mechanism to draw the global dependence between the input and output. In the calculation, the input needs to be linearly transformed to obtain the matrices: Query (Q), Key (K), and Value (V). As expressed in Equation (7), the calculation formula can be written as

(7) $$\mathrm{Attention}\left(A,K,V\right)=\mathrm{softmax}\left(\frac{{\mathrm{QK}}^{T}}{\sqrt{d_{k}}}\right)V$$

where $d_{k}$ is the number of columns of the matrix Q and K.

The calculation process of the self-attention mechanism is shown in Figure 3b and its steps are as follows:

Step 1: Create three vectors. The input feature map is linearly projected into three different spaces, resulting in three new vectors, namely Q, K, and V.

Step 2: Calculate the score.

Step 3: Divide by the scaling factor. The score in Step 2 divided by the scaling factor square $\sqrt{d_{k}}$ (the square root of the dimension of K), where the raw attention values are all clustered around the highest scoring value. This step can play the role of scaling and distraction.

Step 4: Normalization by the softmax [63]. The correlation between the current feature vector and each feature vector in the feature graph is obtained by the softmax.

Step 5: Multiply each V vector by the softmax. Reduce the concern of uncorrelated feature vectors.

Step 6: The accumulated weighted value vector generates an updated feature map as output.

Here, since each location has information about other features in the same image, the dependencies between long-distance interval features in space can be obtained. On this basis, the essence of the multi-head self-attention mechanism used in ViT is to split the three parameters Q, K, and V multiple times while the total number of parameters is constant, and each group of split parameters is mapped to different subspaces of high-dimensional space to calculate the attention weight to focus on different parts of the input. After several parallel calculations, the attention information in all subspaces is merged. Due to the different distribution of attention in different subspaces, multi-head self-attention is actually looking for the correlation between the input data from different angles, so that multiple relationships and subtle differences can be encoded. Multiple independent heads pay attention to different information (such as global information and local information) to extract more comprehensive and rich features.

### 2.4. Generator and Discriminator

Due to the introduction of the self-attention mechanism and multilayer perceptron (MLP) structure [64], ViT can reflect complex spatial transformation and long-distance feature dependence, thus obtaining global feature representation. However, ViT ignores local feature details, which reduces the distinguishability between high-frequency information and low-frequency information.

**Figure 4:**
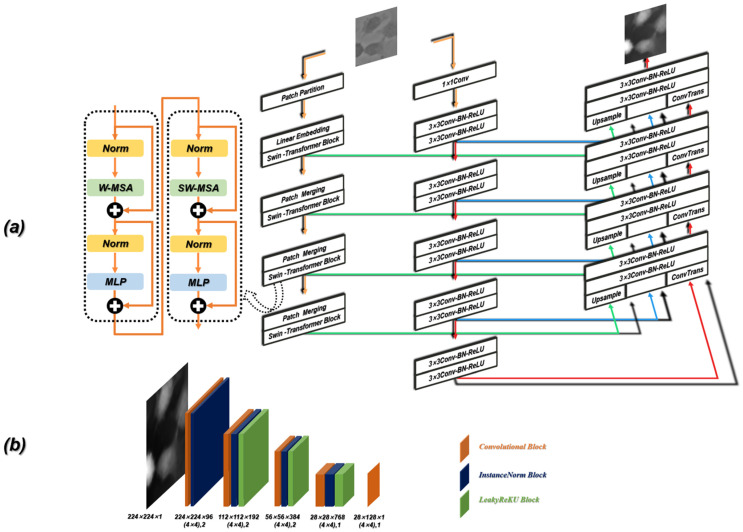
Detailed schematic of the ContransGAN architecture. (**a**) The schematic of the generator. (**b**) The schematic of the discriminator.

**Figure 5:**
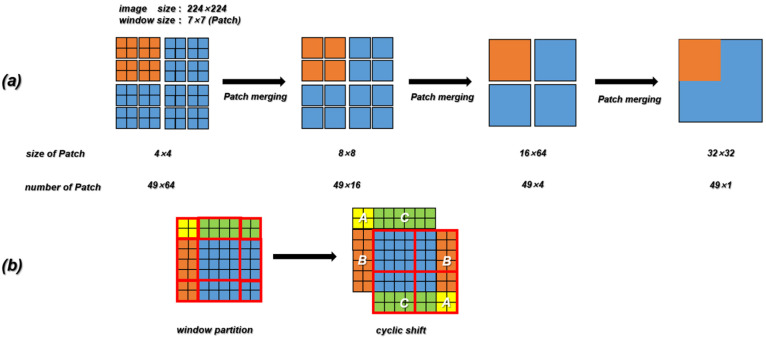
(**a**) The downsampling process of Swin-Transformer. (**b**) Schematic diagram of the SW-MAS operation flow. A, B and C are the regions which need to be moved in the previous feature map.

In our work, the Contrans was proposed as the generator, which uses two sampling channels to combine local features based on CNN and global representation based on Transformer to enhance representation learning. As shown in Figure 4a, the Contrans consists of an improved ViT module branch (termed Swin-Transformer [65]) and a CNN branch. In the process of training, ViT calculates the global self-attention of the feature maps. However, Swin-Transformer is a process in which the window is enlarged, and then the calculation of self-attention is calculated in terms of the window, which is equivalent to introducing the information of local aggregation. This process is very similar to the convolution in CNN, just like the step size and convolution kernel size of CNN, so that the window is not coincident. The difference is that CNN performs convolution calculation in each window, and obtains a new window composed of eigenvalues, which represents the characteristics of this window, while Swin-transformer calculates the self-attention value of each window to obtain an updated window, then merges the windows through the operation of Patch Merging, and continues to calculate the self-attention of the merged window (this process is termed W-MSA), which can also reduce the computational complexity. As shown in Figure 5a, the size of the input is 224 × 224 and the window size is 7 × 7, which is composed of 7 × 7 Patch. A box in Figure 5a represents a window. The size of the Patch changes with the operation of Patch Merging. For example, the Patch of the initial feature map is 4 × 4. By splicing the Patch of the four surrounding windows, the Patch of the feature map of the next layer becomes 8 × 8. By a series of operations, the Swin-Transformer downsampling obtains the feature map with only 1 window and 49 Patch with a size of 32 × 32.

W-MSA operation reduces complexity but brings new problems, that is, a lack of information exchange between windows that are not coincident. In order to exchange information between windows, the region of the feature map can be divided and then moved and spliced. As shown in Figure 5b, the initial feature map is divided into nine regions. We move the upper left region (regions A, B, and C) to the lower right, and then divide the spliced feature map into four equal regions, so that the information between each window can be exchanged. After the downsampling of the Swin-Transformer and CNN branches, the deconvolution operation (transpose convolution) is performed on the feature map obtained by the CNN branch, and the result is spliced with the feature map generated by the corresponding feature layer in the downsampling process. In the process of stitching, to make the size of the three parts of the feature image consistent, the corresponding feature map of the Swin-Transformer needs to be magnified four times by the Upsample operation.

The discriminator in ContransGAN is the PatchGAN [66] structure. As shown in Figure 4b, the input image passes through a ×4 kernel size convolution with stride 2 and the LeakyReLu [67] activation function. The result is the input of the next part. The next part consists of three repeating stages of a convolution layer, a normalization module and a LeakyReLu module. The discriminator divides the input image into overlapping regions, discriminates on each region, and averages the results. The local region of the image is distinguished by the designed discriminator, which improves the ability to model the high-frequency components, so the quality of the image is higher than that of the original GAN’s discriminator.

## 3. Results and Discussion

### 3.1. Results of the Proposed Network

**Figure 6:**
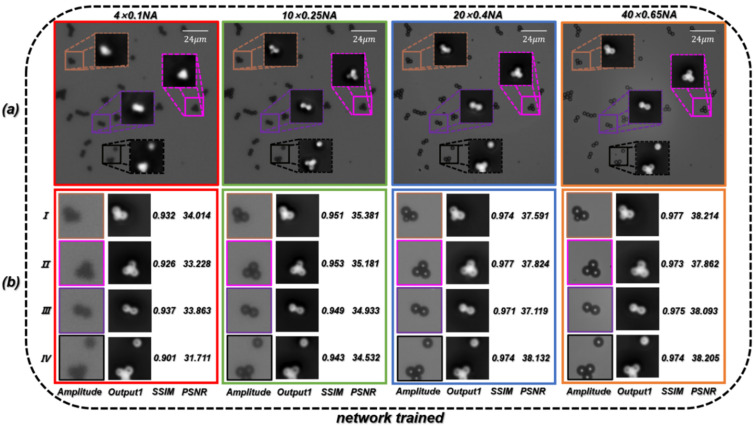
Test results of PSMs by the ContransGAN-All. (**a**) Microscopic images of the same FOV under a different NA objective and the quantitative phase images reconstructed by TIE. (**b**) Results for the corresponding region. Ground truth labels are the quantitative phase images under the 40×/0.65 NA objective in (**a**); SSIM values and PSNR reflect the quantitative relationship between ground truth labels and Output1.

**Figure 7:**
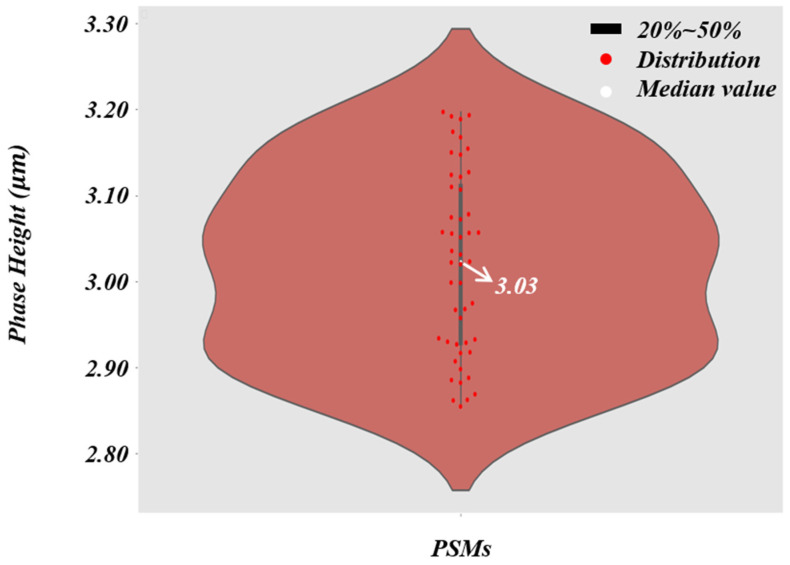
The violin plot of the PSMs’ phase height. The thick black line in the middle indicates the interquartile range, the thin black line extending from it represents the 95% confidence interval, the white dot is the median value, and the red spots indicate the distribution of the PSMs’ phase heights.

According to the formula of resolution [68], the theoretical resolution of the 4×/0.1 NA objective is 2.75 µm. In order to directly reflect the super-resolution effect of the trained network, we first used PSMs with a diameter of 3 µm as specimens. As shown in Figure 6a, the resolution of the reconstructed phase image can be gradually improved by converting a microscope objective with a larger NA. When using the microscopic images captured by the 40 ×/0.65 NA objective, the quantitative phase images of PSMs with HR and accurate surface morphology can be recovered. We used the microscopic images obtained by the four different NA objectives as the training dataset to create $R_{A}$. The trained network is termed ContransGAN-All. In order to quantitatively evaluate the test results, we used the scale-invariant feature transform (SIFT) [69] algorithm to obtain the ground truth labels matching the output by the original HR quantitative phase images, and then calculated the structural similarity (SSIM) [70] values and peak signal-to-noise ratio (PSNR) [71] between the output images and the ground truth labels. The test results of the network are shown in Figure 6b and the results show that ContransGAN-All can accurately reconstruct the corresponding HR quantitative phase images for different resolution microscopic images, and the SSIM values between the output images and the corresponding ground truth labels are more than 0.90 and the PSNR values are greater than 31 dB. It preliminarily proves that the proposed network framework can directly generate the corresponding high-quality HR quantitative phase images through the LR microscopic images. Moreover, with the improvement of the resolution of the input images, the quality of the output images gradually improves. This is mainly because for ContransGAN-All, the higher the resolution of the input images, the richer the detailed structure information they contain, and the more features can be extracted to establish the mapping relationship between the LR microscopic images and the HR phase images to constrain the network to achieve better results. In addition, to prove that the ContransGAN is quantitative, we randomly calculated the phase heights of 50 PSMs generated by the ContransGAN-All. As shown in Figure 7, the result shows that the phase heights of these generated PSMs are all in the range of 2.8 µm–3.2 µm, corresponding to a median value of 3.03 µm, which is consistent with expectations (the average relative error is less than 6%).

**Figure 8:**
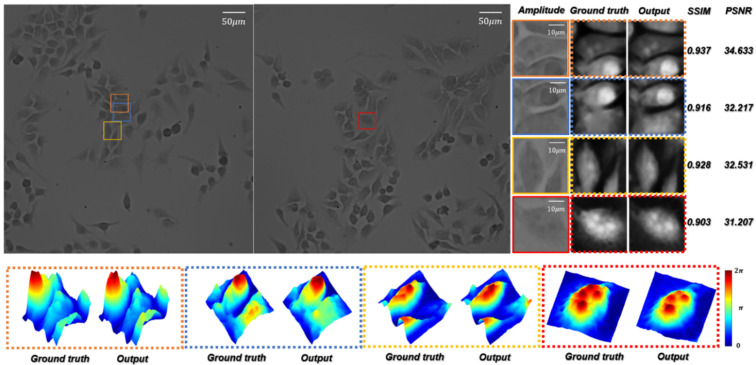
Test results of Hela cells by the ContransGAN-Hela. Amplitude represents the LR microscopic images; ground truth represents the HR quantitative phase images reconstructed by TIE; output represents the output images of the ContransGAN-Hela; SSIM and PSNR reflect the quantitative relationship between the ground truth and output; the dotted frame below is the three-dimensional visual phase distribution in the corresponding FOVs.

In order to test the HR quantitative phase images’ generation quality of the network for biological samples in optical imaging tasks, we used the microscopic images of Hela cells by the 10×/0.25 NA objective as the training dataset to create $R_{A}$. The trained network is termed ContransGAN-Hela. As shown in Figure 8, the SSIM values between the output images and the corresponding ground truth labels of the test results are all above 0.90 and the PSNR values are also greater than 31 dB. Comparing the amplified output images with the ground truth labels, it can be easily found that the ContransGAN-Hela can accurately perceive the high-frequency information in the LR intensity image, establish the mapping relationship between the microscopic images and the quantitative phase images, and give feedback on the output images. Therefore, the proposed ContransGAN is also robust for biological samples, which usually have a complex structure. In order to intuitively compare and analyze the image quality generated by the ContransGAN, we calculated the average SSIM value and PSNR between all generated HR quantitative phase images and the corresponding ground truth labels (Table 1). It can be concluded from the table that the imaging quality of PSMs with a relatively simple structure is better than that of complex biological samples. The main reason is that in the training process of the two networks, in order to ensure the consistency of the network training process, we did not change any parameters. Therefore, only the training images affect the final network performance, so the complexity of the images in the training data determines the quality of the network output image. The standard deviations (std) of SSIM and PSNR of different types of specimens indicate that the more complex the image information is, the more variables there are between the input and output, and the more difficult it is to establish the mapping relationship.

### 3.2. Comparison of Network Performance

**Figure 9:**
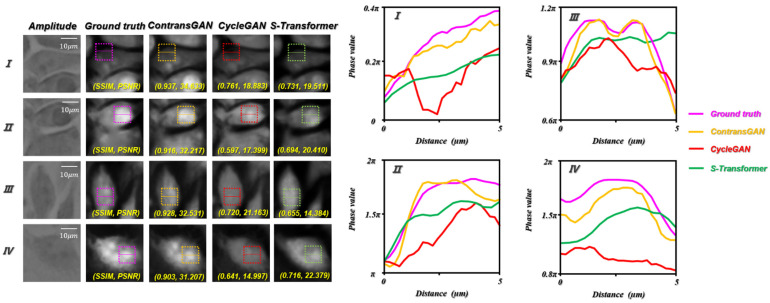
Test results of Hela cells by the CycleGAN-Hela and S-Transformer. SSIM and PSNR reflect the quantitative relationship between the ground truth and the network output phase images; the curve on the right is the phase value curve of the realization part in the dotted line box in the corresponding FOV. I, II, III and IV are different ROIs.

In this paper, compared with the CycleGAN, which uses U-Net [34] as the generator, the difference between the proposed ContransGAN and the CycleGAN is that in order to improve the feature extraction ability of the model, we propose a new generator, termed Contrans. To compare the performance of the modification, we trained the CycleGAN (U-Net as the generator) and S-Transformer (Swin-Transformer as the generator) with the same training dataset. The other hyperparameters, including the learning rate, learning epoch, and batch size, are the same as the ContransGAN-Hela. The results are shown in Figure 9. Compared with the SSIM values and PSNR in Figure 8, the quantitative phase images reconstructed by ContransGAN-Hela are more accurate and have a better image quality. Although CycleGAN and S-Transformer can output a phase image that looks similar in structure, their phase distribution is inaccurate and some areas of the image are distorted. Specifically, in terms of detail generation, ContransGAN-Hela can extract the features of the LR microscopic images as much as possible, so that the final generated quantitative phase images are close to the real distribution. However, CycleGAN and S-Transformer only use CNN or Transformer for feature extraction and cannot fully utilize the information in LR microscopic images, so the generated quantitative phase images lose many detailed features. For further comparison, we plotted the normalized phase distribution curve along the implementation part in the dashed box. It is obvious that the yellow curve output by ContransGAN-Hela matches the purple curve of the ground-truth label image, although in Figure 9 IV, since there is low contrast in the LR microscopic images, the final result has some deviation from the real distribution, but the phase distribution is almost the same. Relatively speaking, there is a considerable error between the red curve and the curve output by CycleGAN and S-Transformer.

### 3.3. Generalization Capability and Accuracy Analysis

**Figure 10:**
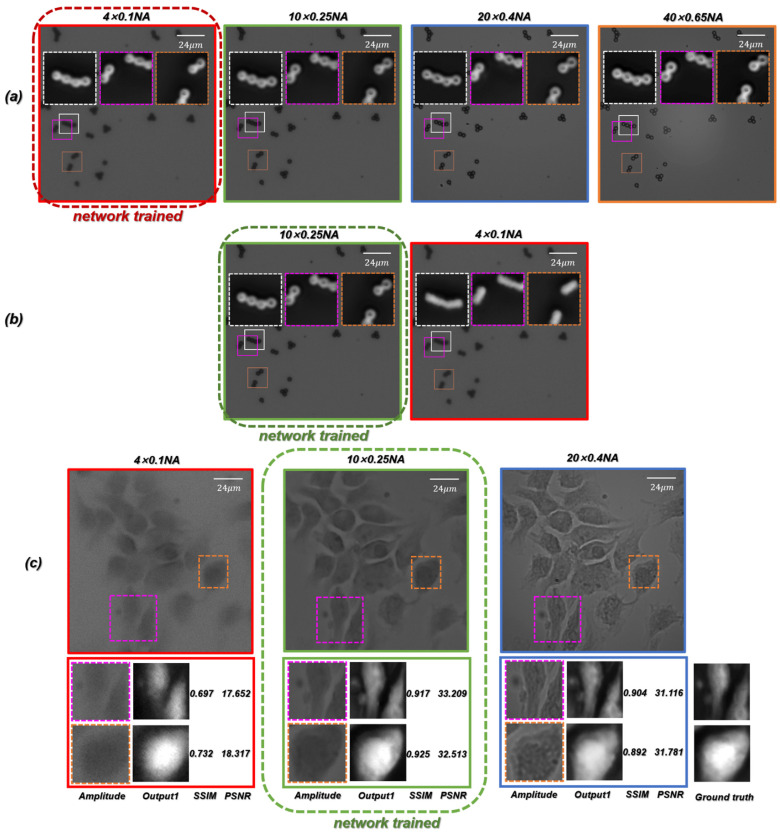
Test results of microscopic images captured by different NA objectives using the ContransGAN trained by microscopic images captured by a certain NA objective. (**a**) The network trained with the microscopic images under the 4×/0.1 NA objective generated HR phase images of the microscopic images captured by the higher NA objective. (**b**) The network trained with the microscopic images under the 10×/0.25 NA objective generated HR phase images of the microscopic images captured by the higher NA objective. (**c**) The network trained with the microscopic images under the 10×/0.25 NA objective generated HR phase images of the microscopic images captured by the other NA objectives.

The above discussion is based on the microscopic images training network obtained under the objective containing different NA. In order to further test the generalization performance of the proposed ContransGAN, we used only the microscopic images of PSMs captured under the 4×/0.1 NA objective, the microscopic images of PSMs captured under the 10×/0.25 NA objective, and the microscopic images of Hela cells obtained under the 10×/0.25 NA objective as training data to train the ContransGAN and obtained three corresponding trained networks. Their corresponding test results are shown in Figure 10. As shown in Figure 10a, the trained network of PSMs captured by the 4×/0.1 NA objective was tested by the other microscopic images captured with different NA objectives. It is obvious that the proposed network can reconstruct high-quality quantitative phase images with good forward compatibility. However, there is no good performance of the network backward compatibility. As shown in Figure 10b,c, the network that was trained with the microscopic images captured by the 10×/0.25 NA objective was tested by the microscopic images captured with the smaller NA objective (4×/0.1 NA). The results show that the network cannot be backward compatible to generate high-quality HR quantitative phase images. It is not difficult to understand that when training with LR microscopic images, the features extracted by the network to establish the mapping relationship between images also exist in the corresponding HR microscopic images with richer information, so the network trained with LR microscopic images can be better reconstructed to generate HR quantitative microscopic images when using HR microscopic images as the network input. Conversely, the network trained by the HR microscopic images with richer image information cannot reflect the corresponding mapping relationship because of the lack of detail features in the LR microscopic images, so the generated images have only approximate morphological features. Especially when imaging biological samples with a relatively complex structure, the network trained by the HR microscopic images is used to generate quantitative phase images of LR microscopic images, which will also be affected by the noise in the original LR microscopic images. In our work, the HR quantitative phase images corresponding to microscopic images with different resolutions can be quickly generated by the trained ContransGAN, which trained with the microscopic images captured under the 4×/0.1 NA objective.

**Figure 11:**
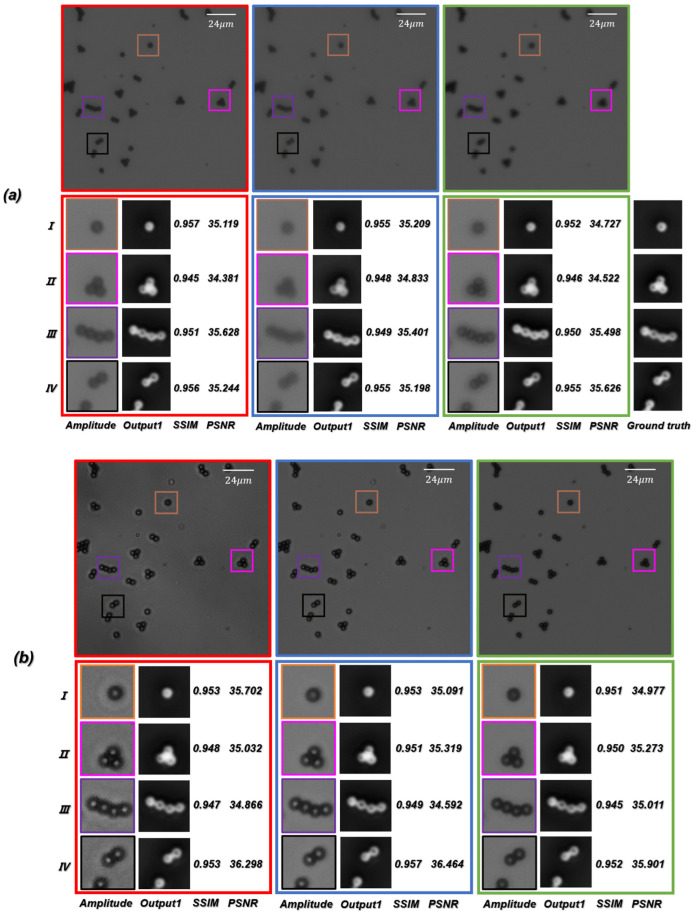
(**a**) Test results of the LR out-of-focus microscopic images. (**b**) Test results of the LR microscopic images with different contrast captured at different apertures of the concentrator.

In practical optical imaging tasks, it is difficult to stay in-focus during long-term observation or imaging. We need to consider the performance of the network if an object is located at distances different from those in the training dataset. In order to further test the generalization performance of the ContransGAN, we trained the network with the out-of-focus microscopic images with an interval of 550 μm between −10 μm and 10 μm from the focal plane under the 10×/0.25 NA objective. Then, we tested the trained network with the out-of-focus microscopic image captured at any distance from −10 μm to 10 μm under the same objective and compared the generated results with the ground truth labels. As shown in Figure 11a, the results indicated that the trained network is able to correctly obtain the mapping relationship between LR out-of-focus microscopic images and the corresponding in-focus HR quantitative phase images with the values of SSIM versus PSNR being above 0.94 versus 34 dB, respectively. This means that the proposed ContransGAN can perform auto-focusing, phase retrieval, and super-resolution imaging at the same time.

Since phase retrieval through TIE requires capturing of microscopic intensity images at the aperture of the diaphragm S≈0.3, and the acquisition of the LR microscopic images requires constant switching of the aperture of the concentrator, it is natural to consider how well the network can perform if the test microscopic images are captured with different apertures of the concentrator. To test this, we trained the ContransGAN with the microscopic images with different contrast obtained by different apertures of the diaphragm under the 10×/0.25 NA objective. We tested the trained network with the microscopic images captured under the same objective at any aperture of the concentrator, and also compared the generated results with the ground truth labels. As shown in Figure 11b, the results indicated that the trained network is able to correctly give the mapping relationship between LR microscopic images different contrast and the corresponding HR quantitative phase images, with the values of SSIM versus PSNR being above 0.94 versus 34 dB, respectively. This proves that even if the contrast of the LR microscopic intensity images is not systematic, the proposed ContransGAN is robust and can provide an accurate prediction.

## 4. Conclusions

In summary, we introduced a novel end-to-end deep learning-based network framework for super-resolution QPI. It can recover the corresponding HR quantitative phase image from an LR microscopic intensity image captured by a commercial microscope. The framework does not need to train with paired data. Using the proposed Contrans as the generator, the feature extraction ability of the network is greatly enhanced and the information in the LR microscopic images can be fully utilized. After training, the HR quantitative phase information of the object can be quickly extracted from a single LR microscopic intensity image with different resolutions. The feasibility of the proposed framework for QPI was quantitatively proved by experiments. The framework can adapt to various problems in optical microscopic imaging, such as defocus, different resolution, and different contrast, and has strong robustness.

## Figures and Tables

**Table 1:** Quality evaluation index of the test output image of different types of specimens.

Objective	4 × 0.1/NA		10 × 0.25/NA		20 × 0.4/NA		40 × 0.65/NA	
Index	SSIM	PSNR	SSIM	PSNR	SSIM	PSNR	SSIM	PSNR
	(std)	(std)	(std)	(std)	(std)	(std)	(std)	(std)
PSMs	0.933 (±0.0107)	34.032 (±1.0231)	0.952 (±0.0113)	35.214 (±0.9892)	0.975 (±0.0118)	38.963 (±0.9153)	0.976 (±0.0097)	39.331 (±0.8322)
Hela cells	0.898 (±0.0172)	31.751 (±1.2048)	0.922 (±0.0151)	32.171 (±1.1920)	0.939 (±0.0135)	34.751 (±1.0723)	0.943 (±0.0129)	35.380 (±1.0133)

## Data Availability

Data underlying the results presented in this paper are not publicly available at this time but may be obtained from the authors upon reasonable request.
